# Spoken words affect visual object recognition via the modulation of alpha and beta oscillations

**DOI:** 10.3389/fnins.2025.1467249

**Published:** 2025-04-14

**Authors:** Piermatteo Morucci, Francesco Giannelli, Craig G. Richter, Nicola Molinaro

**Affiliations:** ^1^Department of Basic Neurosciences, Faculty of Medicine, University of Geneva, Geneva, Switzerland; ^2^Basque Center on Cognition Brain and Language (BCBL), University of the Basque Country UPV/EHU, San Sebastian, Spain; ^3^Cognition and Brain Plasticity Unit, IDIBELL, L’Hospitalet de Llobregat, Barcelona, Spain; ^4^Department of Cognition, Development and Educational Psychology, University of Barcelona, Barcelona, Spain; ^5^Ikerbasque, Basque Foundation for Science, Bilbao, Spain

**Keywords:** neural oscillations, concepts, categorization, object recognition, electrophysiology, bilingualism

## Abstract

Hearing spoken words can enhance the recognition of visual object categories. Yet, the mechanisms that underpin this facilitation are incompletely understood. Recent proposals suggest that words can alter visual processes by activating category-specific representations in sensory regions. Here, we tested the hypothesis that neural oscillations serve as a mechanism to activate language-generated visual representations. Participants performed a cue-picture matching task where cues were either spoken words, in their native or second language, or natural sounds, while their EEG and reaction times were recorded. Behaviorally, we found that images cued by words were recognized faster than those cued by natural sounds. This indicates that language activates more accurate semantic representations compared to natural sounds. A time-frequency analysis of cue-target intervals revealed that this label-advantage effect was associated with enhanced power in posterior alpha (9–11 Hz) and beta oscillations (17–19 Hz), both of which were larger when the image was preceded by a word compared to a natural sound. These results suggest that alpha and beta rhythms may play distinct functional roles to support language-mediated visual object recognition: alpha might function to amplify sensory representations in posterior regions, while beta may (re)activate the network states elicited by the auditory cue.

## 1 Introduction

Hearing certain natural sounds (e.g., the croak of a frog) appears to automatically activate conceptual knowledge, enabling the perceptual system to quickly identify objects in the surroundings (e.g., the presence of a frog). Learning such cross-modal associations represents a crucial prerequisite for mediating interactions with the environment. In humans, conceptual representations can also be activated via language (e.g., “frog”). However, unlike natural sounds, linguistic symbols are categorical, making them more suited to activate semantic information in a format that transcends within-category differences. It remains unclear whether phylogenetically young systems like language exert effects on perception similar to natural sounds, and what brain dynamics might support such effects.

Conceptual representations activated by auditory cues have been shown to interact with the visual system in different ways. For instance, hearing words and natural sounds can rapidly drive visual attention toward specific entities in a scene (Huettig and Altmann, 2007); facilitate the recognition and discrimination of congruent object categories (Boutonnet and Lupyan, 2015; Edmiston and Lupyan, 2015); lower the detection threshold for ambiguous objects (Lupyan and Ward, 2013; Ostarek and Huettig, 2017); and even cause sensory illusions (Dils and Boroditsky, 2010). While this body of evidence suggests that both linguistic and non-linguistic cues activate content-specific representations, it is less clear whether these cues activate the *same* representations. Studies directly targeting this issue have often reported a “label-advantage” effect, that is, a facilitation when object recognition is preceded by words compared to non-linguistic cues (Edmiston and Lupyan, 2015; Lupyan and Thompson-Schill, 2012). This effect suggests that language provides a particularly powerful tool to enhance visual processing.

To achieve these facilitatory effects on visual perception, linguistic categories could theoretically follow two possible pathways (Simanova et al., 2016). Language might not bias perceptual processes at early levels but rather interact at later semantic or categorical decision-making stages (Firestone and Scholl, 2014; Gleitman and Papafragou, 2005; Klemfuss et al., 2012). On an alternative account, words could affect visual processing by setting categorical priors that alter early perceptual processing (Boutonnet and Lupyan, 2015; Kok et al., 2014; Thierry et al., 2009). Support for the latter account comes primarily from EEG studies showing that better recognition of images preceded by congruent words was associated with modulations of early event-related potentials (ERPs) such as the P1 (Boutonnet and Lupyan, 2015; Noorman et al., 2018) – putatively considered an electrophysiological index of low-level visual processes (Spehlmann, 1965). Yet, these ERP experiments targeted the perceptual consequences of language cues on visual object recognition i.e., they focused on time interval following the visual stimulus. The mechanisms that could explain prestimulus effects of language on visual perception remain largely unknown^1^.

Analysis of oscillatory activity provides an excellent opportunity to study language-driven prestimulus modulations during visual object recognition. Based on previous human and animal studies, candidate mechanisms to carry sensory representations are low-frequency oscillations in the alpha/beta-band (Arnal and Giraud, 2012; Bastos et al., 2012; Bastos et al., 2015; Michalareas et al., 2016). Rhythmic brain activity in these frequency bands has been suggested to play a large variety of roles in top-down processing, which could be crucial to support visual object recognition. For example, alpha synchrony has been associated with filtering of task irrelevant information and enhancement of neural representations during tasks involving attention, prediction, mental imagery and working memory (Hari et al., 1997; Jensen et al., 2002; Mayer et al., 2015; Mo J. et al., 2011). This mechanism could be crucial to speed up object recognition by silencing neural populations encoding irrelevant object categories and activating those linked to the target object. Similarly, beta oscillations have been implicated in perceptual expectations (Arnal and Giraud, 2012), online maintenance of cognitive states (Bressler and Richter, 2015; Engel and Fries, 2010) and (re)activation of task-specific cortical networks (Spitzer and Haegens, 2017). Within the context of visual object recognition, this process can facilitate the activation of network states associated with the target object, thereby speeding up its recognition after the image is presented. Based on these findings, we hypothesized that any object recognition advantage for spoken words over natural sounds would be associated with a difference in cortical alpha/beta dynamics.

In the present study, we used a cue-picture matching task to test the hypothesis that language enhances visual object recognition by setting categorical representations via the modulation of alpha/beta oscillations. In contrast to previous studies, we (i) focused on the time interval preceding the onset of the visual object, targeting top-down signaling directly; and (ii) included words from participants’ first (L1) and second (L2) languages, to assess whether the previously reported label advantage extends to language systems acquired later in development. We hypothesized that, if the label advantage arises because words provide refined categorical representations to the visual system, then any differences in object recognition cued by words vs. natural sounds should be associated with modulations of oscillatory alpha/beta dynamics before the onset of the target picture.

## 2 Materials and methods

### 2.1 Participants

We tested a total of 25 Basque-Spanish bilingual speakers. Note that in earlier studies investigating the label advantage in object recognition, a sample size of 15 participants was sufficient to detect the behavioral label-advantage effect (Boutonnet and Lupyan, 2015). Participants were native speakers of Basque who began acquisition of Spanish after 3 years of age (13 female participants; age range 18–33, mean = 25.66, SD = 5.45, age of acquisition of Spanish = 4.23 y.o., SD = 1.33). All participants were right-handed, with no history of neurological disorders and had normal or corrected-to-normal vision. They received a payment of 10€ per hour for their participation. Before taking part in the experiment, all participants signed an informed consent form. The study was approved by the Basque Center on Cognition, Brain and Language (BCBL) Ethics Committee in compliance with the Declaration of Helsinki. Participants completed several language proficiency tests in both Spanish and Basque (see Table 1). First, participants were asked to self-rate their language comprehension (on a scale from 1 to 10, where 10 is a native-like level). All participants rated themselves as highly proficient in both Basque and Spanish. Participants also performed “LexTALE” (Izura et al., 2014; Lemhöfer and Broersma, 2012), a lexical decision task that tested their vocabulary knowledge. They obtained similarly high scores in both Spanish and Basque. In addition, participants had to name a series of pictures using vocabulary of increasing difficulty in both languages. Here as well, participants achieved native-range scores in both languages. Finally, all participants were interviewed by balanced bilingual linguists who rated them on a scale from 0 to 5: no participants had a score below four in either language.

**TABLE 1:** Measures of linguistic proficiency in Basque (L1) and Spanish (L2).

Measure	Basque	Spanish	*T*-tests results (*t, p*)
Self-evaluation (0–10)	9.04 (0.16)	9.39 (0.24)	–4.7, 0.01
LexTALE Basque (0–50); Spanish (0–60)	46.04 (2.67)	54.09 (4.13)	0.86, 0.39
Picture naming (0–65)	64.19 (1.47)	63.38 (1.62)	1.43, 0.16
Interview (0–5)	5 (0)	4.95 (1.33)	0.15, 0.89

### 2.2 Stimuli

The visual stimuli comprised 50 pictures representing 10 animate (e.g., bird) or inanimate (e.g., camera) object categories. Each of these 10 categories was represented by five different highly recognizable images (.png extension, white background, 2,000 × 2,000 pixels): three color photographs obtained from online image collections, one normed color drawing (Rossion and Pourtois, 2004), and one “cartoon” image (Thierry et al., 2009). We selected different instances for each category in order to provide visual heterogeneity.

The audio stimuli comprised 10 words in Basque (L1), 10 words in Spanish (L2) and 10 natural sounds, each referring to one of the object categories. Both the Basque and Spanish words were recorded by a balanced female Spanish-Basque bilingual speaker to ensure that word comprehension was not influenced by voice or pronunciation style. Natural sound stimuli were downloaded from online libraries. Overall, the mean length of the audio stimuli was 0.8 ± 0.05 s (Word in L2, mean = 0.81 s, SD = 0.21; Word in L1, mean = 0.77 s, SD = 0.23; Natural Sounds, mean = 0.84 s, SD = 0.2). Comparing word durations pairwise across conditions using independent sample *t*-test revealed no significant difference between conditions (Word in L1 vs Word in L2: *p* = 0.46; Word in L1 vs Natural Sounds: *p* = 0.11; Word in L2 vs Natural Sounds: *p* = 0.63).

In order to test that sounds and images were unequivocally identifiable, we asked a group of Basque-Spanish bilinguals (*N* = 20), who did not take part in the main experiment, to view a selection of images and listen to a selection of sounds. They were told to name the visual and audio stimuli they perceived using the first noun that came to mind. For the present experiment, we only chose images and sounds that were identically named by all 20 participants. In total, experimental stimuli included 50 images from 10 categories, 10 words in Basque, 10 words in Spanish, and 10 natural sounds.

### 2.3 Procedure

**FIGURE 1:**
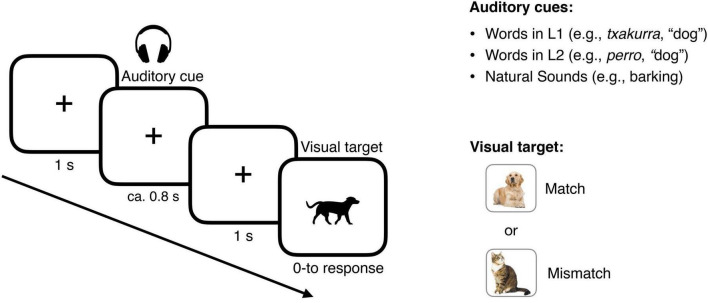
Cue-picture matching task. Participants were presented with auditory cues (words in L1, words in L2, natural sounds) and asked to evaluate whether the subsequent visual target did or did not match the auditory cue.

The EEG experiment was run in a soundproof electrically shielded chamber with dim lighting. Participants sat on a chair, about 60 centimeters in front of the computer screen. Stimuli were delivered using PsychoPy software (Peirce, 2007). We followed the procedure illustrated by Boutonnet and Lupyan (2015). Participants completed a cued-picture recognition task composed of 300 trials (see Figure 1). On each trial, a fixation point appeared at the center of the screen for one second, then participants heard an auditory cue: either a word in L1, (e.g., *igela*, “frog”), a word in L2 (e.g., *rana*, “frog”) or a natural sound (e.g., a croak).

One second after cue offset, a picture appeared on the screen, and participants had to indicate whether the picture did or did not match the auditory cue at the category level by pressing one of two buttons, “yes” or “no,” on the keyboard. The picture remained on screen until the participant responded. The picture matched the auditory cue in 50% of trials (congruent trials); in the other 50%, there was a mismatch (incongruent trials). In the case of incongruent trials, the picture that appeared on screen belonged to a different category. In total, participants were presented with 100 cue-picture pair trials for each condition (Word in L1, Word in L2, Natural sounds), half having a congruent ending and the other half having an incongruent ending. Stimuli presentation was randomized for each participant. The entire experiment lasted 40 min on average.

### 2.4 EEG recording

Electrophysiological activity was recorded from 27 electrodes (Fp1/2, F7/8, F3/4, FC5/6, FC1/2, T7/8, C3/4, CP1/2, CP5/6, P3/4, P7/8, O1/2, F/C/Pz) positioned in an elastic cap (Easycap) according to the extended 10–20 international system. All sites were referenced online to the left mastoid (A1). Additional external electrodes were placed on the right mastoid (A2) and around the eyes (VEOL, VEOR, HEOL, HEOR) to detect blinks and eye movements. Data were amplified (Brain Amp DC) with a filter bandwidth of 0.01–100 Hz, at a sampling rate of 250 Hz. The impedance of the scalp electrodes was kept below 5 kΩ; eye electrode impedance was kept below 10 kΩ.

### 2.5 EEG preprocessing

All EEG data analysis was performed using Matlab 2014 with the Fieldtrip toolbox (Oostenveld et al., 2011)^2^ and R (R Core Team, 2015)^3^. For data visualization, we used Matlab or FieldTrip plotting functions, R and the RainCloud plots tool (Allen et al., 2019). The recordings were re-referenced off-line to the average activity of the two mastoids. Epochs of interest were selected based on cue type (words in L1, words in L2, natural sounds) and congruency (match, mismatch), resulting in six different sets of epochs, computed from –3 to 1.5 s with respect to image onset.

Trials in which subjects provided incorrect responses in the behavioral task were removed from the analysis. Spatial-temporal components of the data containing eye and heart artifacts were identified using independent component analysis and subsequently removed. Overall, we removed an average of 2.14 components per subject. We then identified epochs containing additional “muscle” and “eye blink” artifacts using an automatic artifact detection procedure (z-value threshold = 12). Trials selected as possibly contaminated by artifacts were visually inspected and removed (∼8%). Finally, we removed a few additional trials containing artifacts using a visual inspection procedure (∼0.11%). Three of the 25 initial participants were excluded from the analysis because more than 25% of their trials were rejected, leaving a final sample of 22 participants for the subsequent analysis. After preprocessing, the mean number of trials over participants for the natural sounds, words in L2 and words in L1 conditions was 90.4 (SD = 4.06), 92.18 (SD = 4.28) and 91.04 (SD = 3.95), respectively.

### 2.6 Statistical analysis

#### 2.6.1 Behavior

We used the R environment (version 4.0.0; R Core Team, 2020) and lme4 package (Bates et al., 2014) to perform mixed effect regression on reaction time data, following a procedure similar to that illustrated in Boutonnet and Lupyan (2015). Predicted reaction times (calculated from the onset of the target image until the participant’s response) were computed by fitting the model with cue-type (words in L1, words in L2, natural sounds), congruency (match, mismatch), and their interaction as fixed factors, and by adding by-subject random slopes for the effect of cue type and congruency. Subsequent pairwise comparisons were performed using estimated marginal means (Bonferroni-corrected for multiple comparisons) with emmeans (Lenth et al., 2018). Because no reliable interaction was detected, *post hoc* comparisons were based on a model with the same syntax as the one presented above but excluded the interaction term, in order to facilitate the interpretability of *post hoc* analysis. Accuracy was not analyzed statistically because it was near ceiling (98%). For the analysis of behavioral data, we excluded the same three participants that were excluded from the EEG analysis. Moreover, we excluded all incorrect trials (1.88%), as well as a few trials in which participants’ responses exceeded 3 s (0.28%). These trials were also excluded from the EEG analysis. Reaction times were log-transformed to improve normality.

#### 2.6.2 Spectral power

A time-frequency analysis of artifact-free EEG trials was performed. Before applying spectral decomposition, the latency of each epoch was reduced to –1.5 to 0.5 s with respect to image onset. The time-varying power spectrum of single trials was obtained using a Hann sliding window approach (0.5 s window, 0.05 s time steps) for the frequency range between 0 and 30 Hz, zero-padded to 1 s for a frequency resolution of 1 Hz. We focused on oscillatory activity up to 30 Hz because top-down processes are often associated with oscillations in this frequency band, while higher frequencies are linked to bottom-up processing (Bosman et al., 2012). For the statistical analysis, we computed a single power spectral density estimate for each participant, channel, frequency, and epoch by averaging the spectral estimates centered on the –0.75 to –0.25 s time interval. We selected this time-interval to obtain more accurate spectral estimates, as activity here is largely uncontaminated by activity evoked by the preceding auditory event or subsequent visual stimulus.

#### 2.6.3 Grand-average power spectrum

**FIGURE 2:**
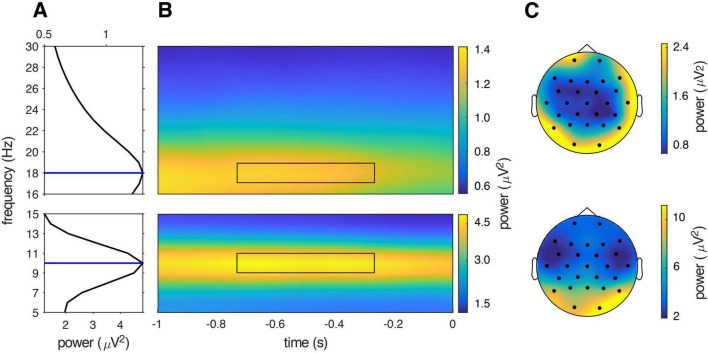
Time-frequency peaks and topographies. **(A)** Alpha and beta peaks in the grand-average raw power spectrum of all epochs across conditions, during the –0.75 to 0.25 s pre-target time interval. The blues lines indicate the raw power peaks as local maxima. **(B)** Time-frequency representation of grand-averaged data for the alpha and beta-band, in the 1 s time window between the offset of the auditory cue (–1 s) and the onset of the image (0 s). The black rectangle denotes the time-frequency interval selected for the statistical analysis. **(C)** Topography of the time-frequency interval of interest.

In order to compute the power spectrum, we combined spectral estimates for congruent and incongruent trials for each cue-type condition, resulting in three different data sets (words in L1, words in L2, natural sounds). Note that time-frequency representations for congruent and incongruent conditions should be indistinguishable during the prestimulus time window since subjects had no way of anticipating the trial type. Spectral estimates were then averaged over trials, participants, channels, and cue-type conditions, resulting in a single value for each of the 30 frequency bins (i.e., the grand-average power spectrum). A peak-finding algorithm was used to identify spectral peaks as local maxima in the grand-averaged power spectrum. Two peaks, one at 10 Hz and one at 18 Hz emerged from this analysis (Figure 2A). Based on these peaks, frequencies of interest (FOI) were obtained as the average of the frequency peaks ± 1 Hz: that is, 9–11 Hz and 17–19 Hz, respectively (Figure 2B). We refer to these band estimates as alpha and beta band power, respectively. The topographical distribution indicates that these frequency peaks were larger over posterior electrodes for both the alpha (electrodes showing the greater effect: O1, O2, P8; mean = 9.91 μV2, SD = 1.88) and beta (electrodes showing the greater effect: O1, O2, P7; mean = 2.39 μV2, SD = 0.1) frequency bands (Figure 2C).

#### 2.6.4 Prestimulus spectral differences between cues

Spectral estimates for each cue-type (words in L1, words in L2, natural sounds) were averaged over trials. To reduce individual differences in overall EEG power, normalization was applied by converting the time-frequency power for each condition into percent signal change relative to the average power over all three conditions and channels, as performed by Bogaerts et al. (2020). This procedure removes individual differences in signal power, without distorting the relative magnitudes of the conditions, i.e., it functions as a baseline correction when an appropriate baseline interval is not available. To test whether time-frequency representations in the prestimulus time window differed across cue types, a non-parametric approach (Maris and Oostenveld, 2007) was selected. For each FOI, we implemented a cluster-based permutation test based on a dependent sample F-test with the spectral data for each type of cue (words in L1, words in L2, natural sounds) as the dependent variable. This approach is equivalent to a one-way ANOVA but allowed us to account for the spatial correlation between electrodes (i.e., no *a priori* region of interest needs to be defined). The minimum number of neighboring electrodes required for a sample to be included in the clustering algorithm was set at 2. The cluster threshold F-value (or t-value) was set at an alpha value at the 85th percentile of their respective distributions. Note that this parameter does not impact the false alarm rate of the test. Rather, it sets a cluster threshold for determining when a sample should be considered as a candidate member of a cluster. Small cluster thresholds usually favor the detection of highly localized clusters with larger effect sizes, while larger cluster thresholds favor clusters with larger spatio-temporal extents but exhibit greater diffusion of the effect (Maris and Oostenveld, 2007). Because alpha and beta rhythms usually emerge at the network level, we selected a relatively large cluster threshold, i.e., capturing what appears to be a more globally distributed effect. The number of permutations for the randomization procedure was set at 100,000. The critical alpha-level to control the false alarm rate was the standard α = 0.05. All resulting *p*-values were Bonferroni corrected for the number of FOIs. For each FOI, one significant cluster was detected. In order to assess the directionality of the effect, *post hoc* non-parametric pairwise comparisons were applied. Specifically, power values for each cue-type condition were averaged over all electrodes belonging to the significant cluster and compared pairwise using paired *t*-tests. The alpha-level for the three *post hoc t*-tests was Bonferroni corrected for the number of comparisons. This procedure was applied to each FOI separately.

## 3 Results

### 3.1 Effect of cues on visual object recognition

**FIGURE 3:**
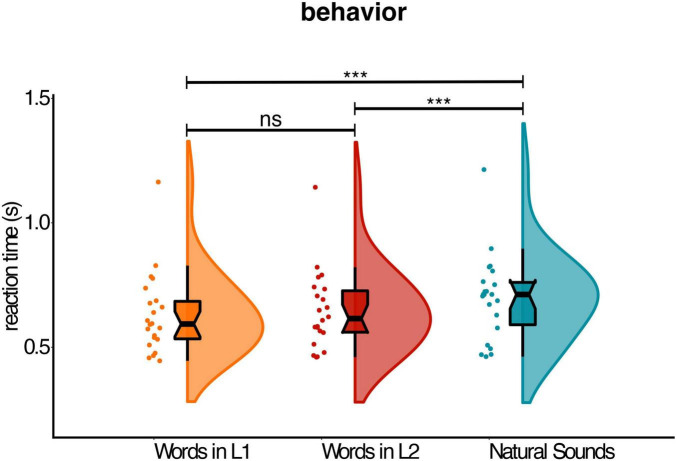
Behavioral results. Mean reaction times (correct trials only) showing the main effect of cue-type on visual object recognition performance. Raincloud plots show probability density. The center of the boxplot indicates the median, and the limits of the box define the interquartile range (IQR = middle 50% of the data). The notches indicate the 95% confidence interval around the median. Dots reflect individual subjects. *** Signifies *p* < 0.001, ns signifies *p* > 0.05.

We first analyzed accuracy. Overall, accuracy was high (98%) and similarly distributed across the three conditions (words in L1 = 98%; words in L2 = 99%, natural sounds = 97%). Participants were clearly at ceiling, so we focused on the analysis of reaction times. Analysis of reaction time responses showed a main effect of Cue-Type [χ^2^(2) = 31.9500, *p* < 0.001] (Figure 3). This was subsequently unpacked via *post hoc* comparisons. Pairwise comparisons using estimated marginal means showed that object images preceded by symbolic cues in both L1 and L2 were identified faster than images preceded by natural sounds (words in L1 – natural sounds: Δ = –0.08, SE = 0.01, *p* < 0.001; natural sounds – words in L2: Δ = 0.06, SE = 0.01, *p* < 0.001). On the other hand, the pairwise effect between words in L1 and words in L2 did not reach the significance threshold (words in L1 – words in L2: Δ = –0.02, SE = 0.01, *p* = 0.06). As in previous studies, we also observed a main effect Congruency [χ^2^(1) = 7.0329, *p* < 0.01], with matching cue-picture pairs leading to faster responses than mismatching pairs. No reliable Cue-Type by Congruency interaction was detected [χ^2^(2) = 1.5310, *p* = 0.46].

### 3.2 Effect of cues on prestimulus alpha rhythms

**FIGURE 4:**
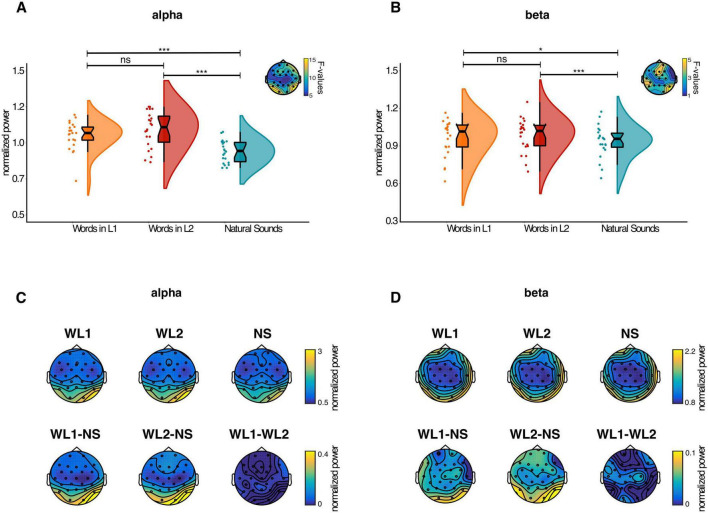
Effect of cues on pre-target alpha **(A)** and beta power **(B)** averaged over the electrodes belonging to the significant cluster and the time-window of interest (–0.75 to 0.25 s pre-target). Conventions for the plot are the same as in Figure 3. Topoplots at the top-right of each figure show the distributions of the F-values and the electrodes belonging to the cluster. At the bottom, topoplots of the normalized time-frequency power for the alpha **(C)** and beta **(D)** bands are shown. The power for each condition is expressed as percent signal change relative to the average power over all three conditions and channels in the time-window of interest (–0.75 to 0.25 s pre-target). The first row shows the topographies for each condition in the time-frequency of interest (time: –0.75 to 0.25 s pre-target; frequencies: 9–11 Hz and 17–19 Hz, respectively). The second row shows the topographical distribution of power differences between conditions. * Signifies *p* < 0.05, *** signifies *p* < 0.001, ns signifies *p* > 0.05.

Differences between spectral power elicited by the three cue-type conditions were assessed using a cluster-based F-test for alpha and beta FOIs separately, focusing on the prestimulus interval. From the analysis of the alpha rhythm, one significant cluster was detected (*p* < 0.01, Bonferroni-corrected for the two FOIs) including several electrodes across the entire scalp (Figure 4A, top-right). The topographical distribution of the F-values is shown in Figure 4A (top-right). To assess the directionality of the effect, spectral power for each type of cue was averaged over all the electrodes belonging to the significant cluster and compared pairwise via *t*-tests. Pairwise comparisons showed that words in L1 and L2 both led to increased alpha power compared to natural sounds [t(21) = 4.57, *p* < 0.001 Bonferroni-corrected; t(21) = 5.48, *p* < 0.001 Bonferroni-corrected, respectively] (Figure 4A). No significant difference was detected between words in L1 and L2 [t(21) = –1.70, *p* = 1 Bonferroni-corrected). Figure 4C below shows the topographical distribution of the normalized power values for each condition, as well as the contrasts between conditions.

### 3.3 Effect of cues on prestimulus beta rhythms

Beta band analysis revealed a pattern of results similar to the alpha rhythm analysis. The cluster-based F-test detected one cluster (*p* < 0.01, Bonferroni-corrected for the number of FOIs) (Figure 4B). The topographical distribution of the F-values, as well as the electrodes belonging to the significant cluster, are shown in Figure 4B (top-right). Spectral power in the beta frequency range was averaged over the electrodes of the significant cluster for each type of cue separately and compared pairwise via *t*-tests. Beta power was larger when images were preceded by words in L1 and L2 compared to natural sounds [t(21) = 2.68, *p* = 0.04 Bonferroni-corrected; t(21) = 4.68, *p* < 0.001 Bonferroni-corrected, respectively], while no significant difference emerged when comparing words in L1 and L2 [t(21) = –1.67, *p* = 0.33 Bonferroni-corrected] (see Figure 4B). Figure 4D below shows the topographical distribution of the normalized power values for each condition, as well as the contrasts between conditions.

## 4 Discussion

Several studies have reported that spoken words can boost visual recognition of object categories, but the neural mechanisms underlying such facilitation are not well established. It has been suggested that effects of language on visual perception arise at early stages of sensory processing; specifically, via the amplification of category-specific representations in sensory regions. In the present study, we investigated the prestimulus effect of language on visual perception, testing the hypothesis that neural oscillations can serve as mechanisms to carry language-generated representations about incoming object categories.

To test this hypothesis, we used EEG to measure prestimulus brain activity and characterize the oscillatory dynamics underlying the label-advantage in object recognition. We reasoned that, if objects are recognized faster because spoken words provide more refined categorical representations than natural sounds, then these cues should differentially modulate prestimulus oscillatory activity in the alpha and beta bands.

We first replicated the previously reported label-advantage and showed that this behavioral effect persisted even when words were presented in a second language. This suggests that verbal symbols deploy more accurate knowledge representations than natural sounds against which incoming inputs can be compared. Importantly, the reported behavioral advantage for spoken words was associated with an increase in the power of alpha and beta rhythms in the time interval between the offset of the cue and the onset of the target object. Such synchronization points to a possible functional role for alpha and beta neural rhythms in the label advantage in object recognition.

While the widespread distribution of significant clusters – including numerous electrodes across the whole scalp – might suggest that the reported effect reflects global alpha/beta network states, the contrasts between conditions indicate that the effect was particularly pronounced over posterior electrodes. This was particularly evident in the alpha frequency-band, suggesting that alpha waves may reflect local oscillatory states originating in occipital regions, in line with findings from the monkey literature (Mo J. et al., 2011).

Enhancement of alpha oscillations in occipital regions has been largely reported when top-down knowledge is directed by a cue toward a specific feature (Bollimunta et al., 2008; Mo J. et al., 2011) or direction (Snyder and Foxe, 2010; Worden et al., 2000). At least two non-mutually exclusive theoretical accounts have been advanced to explain this effect. Some recent proposals posit that enhancement of neural alpha synchronization in task-relevant regions leads to excitatory effects reflecting selective amplification of neural representations of object categories (Klimesch, 2012; Mo J. et al., 2011; Palva and Palva, 2007; Van Kerkoerle et al., 2014), which could in turn facilitate the recognition of the incoming object. For instance, M/EEG studies have reported that alpha power increases in grapheme-processing regions with the predictability of letter identity (Mayer et al., 2015); and in the posterior cortex when meaningful hints precede the discrimination of ambiguous images (Samaha et al., 2018). Similarly, biophysical models indicate that enhancement of prestimulus alpha waves can improve detection performance by increasing the excitation of pyramidal cells, rendering the network state less stable and thus facilitating the activation of a stimulated assembly (Lundqvist et al., 2013). One possibility is that the reported modulations on alpha activity can serve as a mechanism to carry language-generated representations about the structure of visual objects.

Another prominent view is that enhanced alpha power reflects states of inhibition and filtering of task-irrelevant information (Jensen and Mazaheri, 2010; Klimesch et al., 2007). For instance, when attention is directed toward a target on one side of space, posterior alpha-band power increases at electrodes over the hemisphere ipsilateral to the target (Thut et al., 2006; Worden et al., 2000). According to this view, increased alpha oscillations reflect suppression of cortical areas not involved in the task. The alpha effect in our study was right-lateralized and might reflect the inhibition of right-posterior regions to gate sensory information processing to the left-posterior network, where language-perception interactions usually take place (Mo L. et al., 2011). However, this interpretation would also predict alpha desynchronization over left-posterior regions to increase excitability and enhance stimulus processing (Jones et al., 2000; Klimesch et al., 2007). Since we did not find any evidence for the latter effect, we consider it unlikely that alpha synchronization acted as an inhibitory filter in the current study.

A novel result of our study in contrast with similar earlier studies was the differential beta-band modulations that resulted from spoken word vs. natural sound cues. Recent proposals suggest that beta oscillatory activity reflects endogenously driven transitions from latent to active cortical representations of object categories (Spitzer and Haegens, 2017), as well as the binding of neurocognitive network elements associated with a given neural representation (Bressler and Richter, 2015). Under these accounts, beta synchronization provides “a flexible scaffolding that sets up functional neuronal ensembles through temporary synchronization of content-coding cell populations” (Spitzer and Haegens, 2017). In the context of visual object recognition, language-driven beta waves can reactivate neurocognitive networks associated with the target object, enhancing recognition after the image is presented. We speculate that the difference in beta modulations for spoken words vs. natural sounds may reflect a difference in the content of the (re)activated conceptual states – and more importantly, in the amount of retrieved conceptual dimensions, e.g., the size of the neurocognitive network state (Bressler and Tognoli, 2006). Behavioral and eye-tracking experiments have indeed shown that spoken words activate a rich network of features during lexical processing (Huettig and Altmann, 2007). Consequently, processing words might lead to the retrieval of knowledge dimensions that go beyond the purely sensory features of objects, such as conceptual, grammatical, and lexical information. This is partially in line with human and monkey studies showing that beta synchronization carries supramodal information about object categories (Wutz et al., 2018).

We recognize that the interpretations above regarding the role of alpha and beta oscillations should be considered with caution, particularly in relation to specific frequency effects and the distinct roles of alpha and beta rhythms. In fact, the similar power dynamics observed for alpha and beta in this study suggest that they may serve similar functions and support the same underlying mechanisms. The coupling of these frequencies has been previously reported during naturalistic language comprehension, where their power has been shown to similarly encode high-level linguistic computations, such as dependency-building (Zioga et al., 2023). A similar pattern of alpha-beta power modulation has also been observed during single-word production, where it has been linked to the retrieval of lexical-semantic information (Piai et al., 2020). Yet, whether these similar alpha-beta power dynamics reflect a single vs distinct mechanism is currently debated, and previous work has highlighted potential differences in functional contribution (Zioga et al., 2024) and cortical origin (Cao et al., 2022), despite similar activation profiles. In the context of our experiment, and given the aforementioned theoretical accounts, we speculate that alpha-beta power might conjunctively support the top-down encoding of visual semantic categories and recruitment of their respective networks, which is larger for cues activating more precise visual representations. Yet, future studies are needed to assess the specific roles of alpha and beta oscillations in supporting language-mediated visual object recognition, and elucidate whether these frequencies are acting in conjunction or reflecting different mechanisms.

Despite the present findings remain inconclusive about the specific functional roles of alpha and beta oscillations, as well as their potential dissociation, they provide novel contributions to debate on whether language shapes perception at the early or late stages of perceptual processing. Evidence for the former account comes primarily from EEG studies showing that language affects visual processes by modulating early ERP components such as the P1 (Boutonnet and Lupyan, 2015; Noorman et al., 2018) and N170 (Landau et al., 2010). However, studies focusing on post-stimulus activity are also coherent with a later semantic or decision-making account. Indeed, post-stimulus differences, even if very early, could still emerge from rapid feed-forward integration of visual and linguistic information (Thierry et al., 2009). By showing language-induced modulations of alpha-beta power in posterior regions before image presentation, our findings align with the idea that linguistic influences on visual perception arise at early stages and in a top-down manner.

Finally, our study included a novel manipulation not considered in previous studies on categorization: the inclusion of L2 words as auditory cues. Our participants were highly proficient Basque-Spanish bilinguals, with comparable levels of proficiency in both languages, who had acquired their L2 later in development. The effect of top-down processing in bilinguals is currently debated, and largely dependent on factors like proficiency (Kaan, 2014) and age of acquisition (Molinaro et al., 2017). Although it is commonly believed that bilinguals access a semantic system common to both languages (Caramazza and Brones, 1980), recent studies have suggested that top-down processing may be reduced in a second language because of reduced access to perceptual memory resources (Hayakawa and Keysar, 2018), which are known to play an important role in the generation of visual expectations (Hindy et al., 2016). We found comparable behavioral and neural responses after L1 and L2 words cuing visual object recognition. This result is in line with the idea that both languages provide similar types of top-down guidance to the visual system.

However, our results show that L1 and L2 words both affect visual processing differently than natural sounds, challenging the hypothesis that such cues provide similar top-down semantic information to visual regions. Why do symbolic cues enhance visual object recognition performance more than natural sounds? It has been proposed that symbols are extremely effective in compressing semantic information in a format that transcends within-category differences, thus leading to the amplification of those prototypical features that are relevant for distinguishing between exemplars of different categories (Lupyan and Thompson-Schill, 2012). By contrast, natural sounds are primarily linked to context-specific sources (e.g., the barking of a dog may trigger the representation of a specific exemplar of a dog), thus being less effective at cueing categorical states (Edmiston and Lupyan, 2015). Interestingly, ascribing labels to experiences has also been shown to enhance other cognitive functions, such as the retention of items in visual working memory (Souza and Skóra, 2017), learning novel categories (Lupyan et al., 2007), and perceptual categorization across sensory modalities (Miller et al., 2018). These findings indicate that language acts as a powerful tool for compressing information, facilitating different operations important to a multitude of human cognitive processes (Clark and Toribio, 2012). Future studies should investigate whether similar oscillatory mechanisms are employed to support these language-augmented cognitive functions.

## Data Availability

The raw data supporting the conclusions of this article will be made available by the authors, without undue reservation.

## Footnotes

1 Here and throughout the paper, we use the term “prestimulus” to refer to the time interval between the end of the auditory cue and the onset of the visual input.

2
http://www.fieldtriptoolbox.org

3
http://www.r-project.org
